# Muscle-specific strength and its functional correlates in patients with osteosarcopenia

**DOI:** 10.1007/s41999-025-01256-2

**Published:** 2025-06-11

**Authors:** G. Şengül Ayçiçek, E. Caran Karabacak, B. Gökçe, E. Oktay Oğuz, M. Koca, P. Ünsal

**Affiliations:** 1 Department of Geriatrics, University of Health Sciences, Etlik City Hospital, Ankara, Türkiye; 2 Department of Geriatrics, Etlik City Hospital, Ankara, Türkiye; 3 Department of Geriatrics, Yıldırım Beyazıt University, Ankara, Türkiye

**Keywords:** Osteosarcopenia, Muscle-specific strength, Sarcopenia, Osteoporosis, Hand grip strength, Ultrasound, Functional performance, Timed up and Go

## Abstract

**Methods:**

One hundred seventy patients were enrolled in this study and 141 patients were analyzed. Hand grip strength (HGS) was measured with Jamar dynamometer, muscle mass was measured with ultrasound, and sarcopenia diagnosis based on low muscle strength and low muscle mass. Muscle specific strength is calculated as HGS to muscle thickness (kg/cm) and cross-sectional area (kg/cm^2^). Osteoporosis is diagnosed if the T score was below − 2.5 or fragility fracture is present. The patients with both osteoporosis and sarcopenia were accepted as osteosarcopenia.

**Results:**

Mean age of the patients was 76.86 ± 6.74 and 121 (71.2%) were female. Ten percent of the patients was osteoporotic, 25.9% was sarcopenic and 27.1% was osteosarcopenic. In patients with osteosarcopenia, HGS/ Rectus femoris thickness (kg/cm) was 11.35 (2.47–37.5); HGS / Rectus femoris cross sectional area (kg/cm2) was 5.16 (0.82–27.3) and HGS/ Quadriceps femoris thickness (kg/cm) was 5.4 (1.43–13.04). When compared to control, osteoporosis and sarcopenia groups, muscle specific strength was lower in the osteosarcopenia group (p < 0.001, p = 0.009, p < 0.001, respectively). Linear regression analyses revealed that muscle specific strength was significantly associated with timed up and go (TUG) (β = − 0.236, p = 0.030) and L1-L4 T score (β = − 0.233, p = 0.032).

**Conclusion:**

Muscle specific strength- especially rectus femoris and quadriceps femoris thickness—is associated with TUG and L1-L4 scores in osteosarcopenia patients.

## Key summary points

**Aim:**

Comparison of muscle-specific strength in patients with osteoporosis, sarcopenia, and osteosarcopenia and to examine its relationship with functional performance and bone mineral density.

**Findings:**

Muscle-specific strength, particularly when normalized to rectus femoris and quadriceps femoris thickness, is significantly reduced in patients with osteosarcopenia. Moreover, was independently associated with both functionality and T scores.

**Message:**

We need to support the clinical utility of muscle-specific strength as a biomarker for identifying high-risk individuals and tailoring interventions aimed at improving mobility and reducing fracture risk.

## Introduction

Aging is accompanied by a decline in both bone and muscle health, leading to conditions such as osteoporosis and sarcopenia. Osteoporosis is characterized by low bone mass and microarchitectural deterioration of bone tissue, increasing the risk of fractures [1]. Sarcopenia, defined by the progressive loss of skeletal muscle mass and function, contributes significantly to frailty, disability, and mortality in older adults [2]. The concept of osteosarcopenia, the coexistence of osteoporosis and sarcopenia, has recently gained attention due to its cumulative negative effects on mobility, balance, and overall health status [3, 4].

Muscle strength has come to forefront, as it has been shown to be a better predictor of clinical outcomes than muscle mass [5]. Particularly hand grip strength (HGS), has emerged as a reliable indicator of overall muscle function and predictor of clinical outcomes such as falls, hospitalization, and mortality [6]. However, HGS alone may not adequately reflect true muscle function, particularly in the presence of changes in muscle size. Therefore, the concept of muscle-specific strength—the force produced per unit of muscle mass or cross-sectional area—has been proposed as a more precise and informative metric [7]. There are several research in recent years to clarify the diagnostic criteria. The Global Leadership Initiative on Sarcopenia (GLIS) introduced the muscle specific strength to literature in 2024 [8]. GLIS emphasized that Muscle specific strength should be considered as a part of the conceptual definition of sarcopenia. The units of muscle specific strength measures are the ratio of strength to muscle quantity and depend on the exact measures used, examples include Newton/cm2, Newton/kg, etc. [9].

Ultrasound is a reliable, easy to perform on bedside and a quick measurement technique, besides other radiological imagining methods [10]. In Turkish older adults, muscle mass measured with ultrasound predicts sarcopenia as well as fat free mass index [11]. US has distinct advantages over other available modalities for muscle mass because the tool allows for separate measurements of individual muscle groups. the European Geriatric Medicine Society sarcopenia group published a consensus protocol for using US to assess muscle mass [12].

This study aimed to compare muscle-specific strength in patients with osteoporosis, sarcopenia, and osteosarcopenia and to examine its relationship with functional performance and bone mineral density.

## Materials and methods

### Patient characteristics

This cross-sectional study was conducted in a geriatric outpatient clinic. A total of 170 patients aged 65 years and older were initially recruited. Following the application of exclusion criteria, including acute illness, neurological diseases affecting mobility, and incomplete data, 141 participants were included in the final analysis. Patients were categorized in four groups as osteoporosis, sarcopenia, osteosarcopenia, and control. Comprehensive geriatric assessment was performed for all participants.

### Assessment of muscle strength and mass

Muscle strength was evaluated using hand grip strength (HGS), measured with a Jamar hydraulic hand dynamometer in a seated position, with the shoulder adducted, elbow flexed at 90° and the forearm in a neutral position. Measurements were taken three times for each hand with resting intervals, and the highest value was used for the analysis. The thresholds of 16 kg for women and 27 kg for men were used, as recommended by the EWGSOP-2 [2].

Muscle thickness and cross-sectional area of the rectus femoris and quadriceps femoris were measured using B-mode ultrasound imaging. The US evaluations were performed using a 5–12 MHz linear probe (Logiq 200 Pro, and Logiq P5 GE Medical Systems), with participants in a supine position and muscles at rest. Rectus femoris was evaluated at 50% between the anterior superior iliac spine and the superior pole of the patella, in a lying position with knees in 10°, for muscle thickness and muscle cross-sectional area and Quadriceps femoris was evaluated at 30% (proximal or distal was not described) between the anterior superior iliac spine and the proximal end of the patella.To assess the intraobserver and interobserver reliabilities of the measurements, ultrasonographic evaluation of 10 randomly selected patients was repeated by the same observer and a second observer 24 h after the index ultrasonography. The researcher who carried out the ultrasonographic evaluation had 5 years of experience in ultrasound. Muscle-specific strength (MSS) was calculated as the ratio of HGS to muscle thickness (kg/cm) and cross-sectional area (kg/cm^2^).

### Assessment sarcopenia and osteoporosis

Sarcopenia was diagnosed according to the European Working Group on Sarcopenia in Older People 2 (EWGSOP2) criteria, requiring both low muscle strength and low muscle mass [2]. Muscle mass was measured from the demonstrated measurement points in SARCUS Project [12] and cut off values were evaluated from the study of Ozturk et al. [11]. Osteoporosis was diagnosed based on dual-energy X-ray absorptiometry (DXA) with a T-score below -2.5 at the lumbar spine or femoral neck, or the presence of a fragility fracture. Osteosarcopenia was defined as the coexistence of both osteoporosis and sarcopenia.

### Assessment of functionality

Functionality was assessed using the Timed Up and Go (TUG) test. In TUG test, participants were instructed to stand from a chair by sitting position without assistance of upper limb, and to walk for 3 m at usual gait speed, then to turn back in a straight line and to sit back on the chair [13]. The time taken to complete the procedure is calculated and the cut-off value is ≥ 20 s for TUG test.

### Statistical analysis

Statistical analyses were performed using IBM SPSS Statistics 23. Participants were categorized into four groups: normal, osteoporosis, sarcopenia, and osteosarcopenia.

The distribution of continuous variables was assessed using the analytical and visual methods. For normally distributed variables, comparisons between the four groups were made using one-way analysis of variance (ANOVA), followed by post hoc Tukey tests for pairwise comparisons. For non-normally distributed variables, the Kruskal–Wallis test was used, followed by Bonferroni correction for post hoc analysis. Categorical variables were compared using the Chi-square test. Correlations between TUG and muscle specific strength were evaluated using Spearman's correlation coefficient. Multivariate linear regression analysis was performed to assess the independent association between muscle specific strength and functionality. The normality and homoscedasticity of residuals were checked using residual plots and analytical and visual methods. A *p-value* < 0.05 was considered statistically significant.

## Results

The mean age of participants was 76.86 ± 6.74 years, and 71.2% were female. Prevalence rates were 10% for osteoporosis, 25.9% for sarcopenia, and 27.1% for osteosarcopenia. Patients in the osteosarcopenia group presented the lowest muscle-specific strength across all indices. Significant between-group differences were observed for HGS/rectus femoris thickness, HGS/rectus femoris cross-sectional area, and HGS/quadriceps femoris thickness (p < 0.001, p = 0.009, and p < 0.001, respectively). The demographics and clinical parameters of each group are summarized in Table 1.

**Table 1:** The demographics and clinical parameters of the participants

	Control (n = 34)	Osteoporosis (n = 17)	Sarcopenia (n = 44)	Osteosarcopenia (n = 46)	p
Age	74.18 ± 6.31	78. 24 ± 6.64	75.93 ± 6.75	79.28 ± 6.30^a^	0.010
BMI	32.35 ± 5.85	29.48 ± 5.89	28.70 ± 6.64	27.57 ± 6.31^a^	0.021
HGS	20 (9–33)	20 (5–58)	14.5 (4–24)	10 (3–26)^a,b^	< 0.001
TUG	11 (8–17.8)	11 (6–40)	10.05 (4–63)	13 (8–35.3)^a^	0.018
Femur neck T score	− 0.9 (− 2.1–0.1)	− 2.5 (− 3.7 to − 1.4)^a^	− 0.95 (− 2.2–1.2)^b^	− 2.1 (− 4.3–0.8)^a^	< 0.001
Femur total T score	− 0.8 (− 1.8–0.5)	− 2.5(− 2.8 to − 0.7)^a^	− 0.65 (− 2.1–1.8)^b^	− 1.8 (− 4.2 to − 1)^a^	< 0.001
L1-L4 T score	− 1 (− 2.1–2.1)	− 1.7 (− 3.5 to − 0.3)	− 0.6 (− 2.3–3.9)	− 2.5 (− 3.9–1.9)^a^	< 0.001
RF thickness (cm)	1.19 ± 0.43	1.15 ± 0.36	1.04 ± 0.32	0.97 ± 0.33	0.043
RF_ CSA (cm^2^)	3.38 ± 1.76	3.47 ± 2.07	2.61 ± 1.13	2.31 ± 1.02^a,b^	0.003
Quadriceps femoris thickness (cm)	2.31 ± 0.73	2.05 ± 0.60	2.09 ± 0.53	1.89 ± 0.53^a^	0.046
HGS/ RF thickness	19.07 (5.11–43.4)	17.16 (3.1–38.8)	13.88 (3.39–41.2)^a^	11.35(2.47–37.5)^a^	< 0.001
HGS/RF_CSA	7.84 (1.71–27.97)	6.6 (1.03–20.43)	5.36 (1.41–26.16)	5.16 (0.82–27.3)^a^	0.009
HGS/ Quadriceps femoris thickness	10 (2.73–18.75)	9.05 (1.9–20.89)	6.77 (1.82–14.61)^a^	5.4 (1.43–13.04)^a^	< 0.001

*BMI* Body Mass Index, *HGS* Handgrip Strength, *TUG* Time Up and Go, *RF* Rektus Femoris, *CSA* Cross Sectional Area

ab: intragroup Bonferoni post hoc test value

^a^significant differences to control

^b^significant differences to osteoporosis

Comprehensive geriatric assessment results revealed that, patients in the osteosarcopenia group seemed to be more dependent and more frail in terms of geriatric syndromes (Table 2).

**Table 2:** Comprehensive geriatric assessment test scores of the participants

	Control (n = 34)	Osteoporosis (n = 17)	Sarcopenia (n = 44)	Osteosarcopenia (n = 46)	p
Katz ADL	5 (3–6)	5 (2–6)	5 1–6)	5 (0–6)	0.051
Lawton IADL	7 (1–8)	5 (1–8)	6 (0–8)	4 (0–8)^a^	< 0.001
CFS	4 (2–6)	4 (3–7)	4 (2–7)^a^	5 (3–7)^a^	0.001
MNA-SF	12 (7–14)	11 (7–14)	10 (4–13)^a^	10 (2–14)^a^	< 0.001
Mini cog	3 (0–5)	3 (0–5)	2 (0–5)	2 (0–5)	0.311
PHQ-2	1 (0–2)	2 (0–2)	2 (0–2)^a^	1 (0–2)	0.029
SARC-F	2 (0–7)	2 (0–7)	3 (0–10)	5 (0–10)^a^	< 0.001
Falls	8 (24.2%)	8 (47.1%)	16 (36.4%)	19 (41.3%)	0.332
Insomnia	11 (32.4%)	7 (41.2%)	21 (47.7%)	16 (34.8%)	0.490
Polypharmacy	18 (52.9%)	8 (47.1%)	27 (61.4%)	26 (56.5%)	0.753

*ADL* Activities of Daily Living, *IADL* Instrumental Activities of Daily Living, *CFS* Clinical Frailty Scale, *MNA-SF* Mini Nutritional Assessment Short Form, *PHQ-2* Patient Health Questionnaire-2

a: intragroup Bonferoni post hoc test value

^a^significant differences to control

In multivariate linear regression analysis, muscle-specific strength was significantly associated with TUG performance (β = − 0.236, p = 0.030) and L1–L4 BMD T-score (β = − 0.233, p = 0.032), indicating that higher muscle-specific strength corresponds with better functional mobility and higher bone density (Table 3).

**Table 3:** Lineer regression analysis of muscle specific strength with functionality and osteoporosis

	HGS/QF		HGS/RF		HGS/RF CSA	
	β	*p*	β	*p*	β	*p*
TUG	− 0.218	0.046	− 0.236	0.030	− 0.151	0.171
L1-L4 score	0.193	0.077	0.233	0.032	0.175	0.113

## Discussion

This study aimed to compare muscle-specific strength among patients with osteoporosis, sarcopenia, and osteosarcopenia, and to explore its association with functional performance. Our findings demonstrate that muscle-specific strength, particularly when normalized to rectus femoris and quadriceps femoris thickness, is significantly reduced in patients with osteosarcopenia. Moreover, was independently associated with both functional mobility, as measured by the Timed Up and Go (TUG) test, and bone mineral density (BMD), as reflected by L1–L4 T-scores.

Our results contribute to the growing body of evidence that osteosarcopenia represents a clinically significant phenotype with compounded risks, exceeding those of osteoporosis or sarcopenia alone. Osteosarcopenia is increasingly recognized as a geriatric syndrome involving concurrent deficits in bone and muscle, leading to a heightened risk of falls, fractures, loss of independence, and mortality [4, 14, 15]. In our cohort, 27.1% of patients were classified as osteosarcopenic—a prevalence consistent with previous studies reporting rates between 20 and 35% in community-dwelling and hospitalized older adults [16, 17].

Physiological and functional disturbances, such as decreased muscle mass and strength, slow walking speed, and balance problems, have been reported with both osteoporosis and sarcopenia. However, these issues may be exacerbated in individuals with osteosarcopenia, thereby increasing their risk of falling. Osteosarcopenia is strongly associated with falls and fractures leading to increased mortality risk [18]. Consequently, identifying those suffering from osteosarcopenia has significant clinical implications and can effectively reduce fall risk and prevent bone fractures [19]. In the present study, while the prevalence of fall history was similar across the four groups, it was most commonly observed in patients with osteoporosis and osteosarcopenia.

Morimoto et al. showed the association of muscle specific strength with physical performance and history of falls [20]. Although individual measures such as handgrip strength did not correlate with functionality, muscle specific strength was correlated. They mentioned integration of strength and muscle measurements into MSS may provide a more comprehensive assessment of sarcopenia outcomes. Similarly, MSS is related to functionality in older adults especially rectus femoris and quadriceps femoris in our study.

Ultrasound was utilized in this study to assess rectus femoris and quadriceps femoris muscle thickness and cross-sectional area. This method offers several advantages, including non-invasiveness, cost-effectiveness, and bedside applicability. Recent studies have validated ultrasound measurements of muscle architecture against gold-standard imaging such as MRI and CT, demonstrating strong correlations with functional outcomes [9, 21]. Furthermore, rectus femoris has emerged as a particularly informative site for evaluating lower extremity function in older adults, with its decreased thickness being associated with frailty, mobility impairment, and hospitalization risk [22]. The RF CSA, RF thickness have potential value in assessing muscle in osteosarcopenia and can be applied to routine clinical management of older adults [23]. We have evaluated both rectus femoris and quadriceps femoris muscles and found out that MSS is diminished in osteosarcopenic patients.

Our findings underscore the importance of muscle-specific strength as a determinant of functional performance. The negative association between muscle-specific strength and TUG scores supports the hypothesis that lower muscle quality contributes to reduced mobility and increased fall risk. The TUG test is widely used in geriatric assessments as a predictor of functional independence, hospitalization, and mortality [24]. Reduced muscle-specific strength may partly explain why individuals with osteosarcopenia often perform worse on such functional tests compared to their non-sarcopenic or non-osteoporotic peers.

From a clinical perspective, our findings suggest that assessing muscle-specific strength may offer a more sensitive indicator of functional decline than absolute measures of strength or muscle size. Early identification of reduced muscle quality—especially in the context of low BMD—could enable more timely and targeted interventions, such as resistance training, protein supplementation, and pharmacological treatments. Studies have shown that interventions targeting both bone and muscle (e.g., combined exercise and anti-osteoporotic medications) are more effective than single-modality approaches in improving outcomes in this population [25, 26].

Our study has several strengths, including the use of ultrasound for direct muscle measurement, objective assessment of strength via dynamometry, and the integration of functional outcomes. However, limitations should be acknowledged. The cross-sectional design precludes causal inference, and the relatively small sample size in subgroups may limit generalizability. In our patient population, DEXA was utilized for the diagnosis of osteoporosis; however, routine evaluation of muscle mass using DEXA-derived appendicular lean mass (ALM) was not available in standard clinical practice in our country.

## Conclusion

This study highlights that muscle-specific strength is significantly reduced in patients with osteosarcopenia and is independently associated with both functional performance and BMD. These findings support the clinical utility of muscle-specific strength as a biomarker for identifying high-risk individuals and tailoring interventions aimed at improving mobility and reducing fracture risk. Future longitudinal studies are warranted to explore causal relationships and assess the impact of targeted interventions on muscle-specific strength and related outcomes in osteosarcopenic populations.
